# Step-to-Step Variability in Treadmill Walking: Influence of Rhythmic Auditory Cueing

**DOI:** 10.1371/journal.pone.0047171

**Published:** 2012-10-08

**Authors:** Philippe Terrier

**Affiliations:** 1 IRR, Institut de Recherche en Réadaptation, Sion, Switzerland; 2 Clinique romande de réadaptation SuvaCare, Sion, Switzerland; University of Oxford, United Kingdom

## Abstract

While walking, human beings continuously adjust step length (SpL), step time (SpT), step speed (SpS = SpL/SpT) and step width (SpW) by integrating both feedforward and feedback mechanisms. These motor control processes result in correlations of gait parameters between consecutive strides (statistical persistence). Constraining gait with a speed cue (treadmill) and/or a rhythmic auditory cue (metronome), modifies the statistical persistence to anti-persistence. The objective was to analyze whether the combined effect of treadmill and rhythmic auditory cueing (RAC) modified not only statistical persistence, but also fluctuation magnitude (standard deviation, SD), and stationarity of SpL, SpT, SpS and SpW. Twenty healthy subjects performed 6×5 min. walking tests at various imposed speeds on a treadmill instrumented with foot-pressure sensors. Freely-chosen walking cadences were assessed during the first three trials, and then imposed accordingly in the last trials with a metronome. Fluctuation magnitude (SD) of SpT, SpL, SpS and SpW was assessed, as well as NonStationarity Index (NSI), which estimates the dispersion of local means in the times series (SD of 20 local means over 10 steps). No effect of RAC on fluctuation magnitude (SD) was observed. SpW was not modified by RAC, what is likely the evidence that lateral foot placement is separately regulated. Stationarity (NSI) was modified by RAC in the same manner as persistent pattern: Treadmill induced low NSI in the time series of SpS, and high NSI in SpT and SpL. On the contrary, SpT, SpL and SpS exhibited low NSI under RAC condition. We used relatively short sample of consecutive strides (100) as compared to the usual number of strides required to analyze fluctuation dynamics (200 to 1000 strides). Therefore, the responsiveness of stationarity measure (NSI) to cued walking opens the perspective to perform short walking tests that would be adapted to patients with a reduced gait perimeter.

## Introduction

For walking, human beings produce a series of alternating, rhythmical movements of the trunk and limbs, which result in the forward progression of the body. By alternating the stance and oscillation phases of lower limbs, the body moves over a given distance (step length, SpL) in a given duration (step time, SpT) twice during a full gait cycle (or stride  =  two steps). The resulting speed is the ratio between step length and time (step speed: SpS = SpL/SpT). Theoretically, an infinite combination of SpL and SpT, and hence highly variable gait patterns, could result in a steady speed. On the contrary, it is observed that motor control provides highly consistent gait pattern from one stride to the next, with small residual step-to-step fluctuations [1], [2], [3]. At a given speed, it has been shown that preferred (spontaneously chosen) SpL and SpT coincide with minimal energy expenditure [4], [5]. In an experiment that induced visual perturbations, increased step-to-step variability of SpL has been associated with higher energy cost [6]: the causes are likely twofold: a) an increased number of steps with energetically sub-optimal combination of SpL and SpT; b) increased cost induced by active feedback adjustments to restore optimal gait parameters, that require higher muscular activity [5], [6], [7]. Therefore, it is very likely that –by integrating both feedforward (from internal models) and feedback (from sensory inputs) mechanisms – continuous adjustments of basic gait parameter (SpL and SpT) are performed by motor control in order to produce low step-to-step fluctuations and hence an optimal level of energy expenditure.

Apart energy aspects, motor control must also adjust gait to deal with the inherent instability of bipedial locomotion. It has been shown that lateral stabilization plays a central role in this context [8]: lateral foot placement seems actively controlled step-by-step in order to counteract constitutive instability in the frontal plane. Consequently, Step Width (SpW, i.e. the maximal lateral distance between feet during one step) has been extensively studied as a proxy for global dynamic stability and fall risk [9], [10], [11], [12].

The quantification of step-to-step fluctuations has been realized with classical statistical methods that assess the dispersion of data around central trend, such as Standard Deviation (SD) [10], Root Mean Square (RMS) [6], or Coefficient of Variation (CV, defined as SD/mean) [3]. However, these methods assume that gait parameters are purely random processes with no correlation between successive strides, what is unlikely to be the case, because feedback loops are involved in the regulation mechanisms. As a result, alternative methods have been proposed to quantify the temporal dependences in the time series of gait parameters. For instance, the short-term stationarity of the mean throughout a long recording of gait parameters can by assessed (NonStationarityIndex, NSI [13], [14]). Detrended Fluctuation Analysis (DFA) is also used to describe the statistical persistence that exists across consecutive strides [15], [16]. “Persistence” means that deviations are statistically more likely to be followed by subsequent deviations in the same direction (i.e. persist across subsequent data points). Conversely, “Anti-persistence” means that deviations in one direction are statistically more likely to be followed by subsequent deviations in the opposite direction.

To better understand how humans regulate their steps in order to comply with energy and stability requirements, a possibility is to submit individuals to external constraints and to examine the resulting change in step-to-step fluctuation pattern. Here we wish to introduce two type of constraints, namely temporal constraints, induced by Rhythmic Auditory Cueing (RAC), and speed constraints, induced by motorized treadmill. The synchronization of body movements to external rhythm (auditory-motor coordination) is a remarkable ability of the human brain [17], [18]. Step time modulations driven by RAC have been studied in the context of different clinical disorders, such as head injuries, Parkinson's disease or stroke [19]. It can induce a substantial beneficial effect on gait performance [19], [20]. Motorized treadmills are widely used in biomechanical studies of human locomotion. They allow the performance of a large number of successive strides under controlled environment, with a selectable steady-state locomotion speed. In the rehabilitation field, treadmill walking is used in locomotor therapy, for instance with partial body weight support in spinal cord injury or stroke rehabilitation [2].

We [3], [21], and others [22] observed that RAC deeply modifies statistical persistence of stride time, while fluctuation magnitude (CV) remained unchanged. We concluded that, when the walking pace is controlled by an auditory signal, the feedback loop between the planned movement (at supraspinal level) and the sensory inputs induces a continual shifting of stride time around the mean (anti-persistence), but with no effect on the fluctuation dynamics of the other parameters (stride length and stride speed) [1]. The same anti-persistent pattern has been described by Dingwell and Cusumano for stride speed during treadmill walking [23]. The authors proposed that humans use sub-optimal control to correct stride-to-stride deviations: in order to follow the speed imposed by the treadmill, individuals consistently slightly over-correct small deviations in walking speed at each stride [23]. They also proposed a general model of gait regulation based on redundancy theory (Goal Equivalent Manifold, GEM) and Minimum Intervention Principle (MIP).

Recently, we analyzed the combination of both RAC and treadmill walking [21]. Under this dual constraints condition, we observed that stride time, stride length and stride speed exhibited an anti-persistent pattern. Anti-persistent dynamics may be related to a tighter control: deviations are followed by a rapid over-correction that produces oscillations around target values. Under single constraint condition, while stride speed is tightly regulated in order to follow the treadmill speed, redundancy between ST and SL would likely allow persistent pattern to occur. Conversely, under dual constraint conditions, the absence of redundancy among SL, ST and SS would explain the generalized anti-persistent pattern [21].

In the present study, we further documented step-to-step variability in healthy individuals walking on a treadmill, with and without RAC. In the previous study [21], we assessed statistical persistence and variability (CV) in consecutive strides over 5 min. walking. Here, we re-analyzed the same raw data to study variability in 200 consecutive steps. The methodological goal was to explore whether fluctuation dynamics could be analyzed with shorter walking test in the perspective to analyze gait impaired patients in future studies. In addition to basic spatio-temporal parameters (SpT, SpL, SpS), we also compute step width (SpW). The hypothesis was that SpW and SpW variability would be not modified by RAC, because variability in the frontal plane is related to dynamic stability and regulated separately. Standard deviation (SD) among steps was used as variability index. In addition, an index of the sationarity of local means (NonStationarity Index) was also computed. We hypothesized that anti-persistence induced by RAC should be associated with more stationary gait parameters (less variation of local means among consecutive steps). Finally, we assess the strength of the association among variability indexes, including scaling exponents from previous study [21]. Because GEM model assumed that SpT and SpL are cross-regulated, we assumed that variability indexes, as well as statistical persistence, would be correlated.

## Methods

### Participants

In the present article, we re-analyzed data obtained in a previous study [21]. Please refer to this article to obtain supplementary information about the experimental procedure and data analysis. Twenty healthy subjects (10 females, 10 males) participated in the study. The participants' characteristics were (mean (SD): age 36 yr (11), body mass 71 kg (15), and height 171 cm (9). All participants gave written informed consent to take part in the study. The experimental procedure was approved by the local ethics committee (Commission Cantonale Valaisanne d'Ethique Médicale, Sion, Switzerland), in accordance with the ethical standards in the Declaration of Helsinki.

### Experimental procedure

Treadmill speeds imposed to the subjects were: Preferred Walking Speed (PWS), 0.7× PWS (low speed) and 1.3× PWS (high speed). The sequence of the trials was randomly designed. Each walking trials lasted 5 m30: 30 s of habituation to the speed, and 5 min of measurement. Then, the trials with “metronome” condition were performed at the same speeds as the first 3 trials. The imposed cadences were the preferred cadences, which were measured during the first trials without metronome.

### Instrument

The instrumented treadmill was a FDM-TDL (Scheinworks/Zebris, Schein, Germany). The sensor surface contained 7168 (128×56) pressure sensors on a 108.4×47.4 cm grid (1.4 sensors per cm2). Pressure data were sampled at 100 Hz. The raw data consisted for each trial in 30′000 frames of 56×128 points: they were exported for subsequent analysis with Matlab (Mathworks, USA).

### Data analysis

The first 90 sec of each test was not analyzed in order to avoid starting effect. A constant number of 200 steps (100 gait cycles) was kept for the subsequent analysis. The time series of Step Time (SpT), Step length (SpL) and Step Speed, (SpS = SpL/SpT) were obtained by specifically detecting the heel strikes in the frames, and hence calculating time and distance between consecutive steps. From the raw data corresponding to the selected 200 steps, continuous (100 Hz) trajectory of the center of pressure was computed as the weighted average of pressure data, with the standard method for determining barycentre (Sum of mass*position)/(Sum of mass). By using a peak detection algorithm, Step Widths (SpW) were assessed as the maximal left/right distance (medio-lateral signal) reached at each step by the pressure centre.

Central tendencies and fluctuation magnitudes among individuals (N = 20) and conditions (N = 3×2) were assessed by computing Means and Standard Deviations of the time series (M and SD, N = 200). We were also interested to characterize the local, time dependent, fluctuations in the series, irrespectively of the global fluctuation magnitude. Therefore, we computed NonStationarity Index (NSI), which estimates the dispersion of normalized local means [13]: each series is first standardized (by subtracting mean and dividing by SD); then the standardized series is divided in 20 segments of 10 steps; the local average of each segment is computed; the NSI is the SD of these 20 means. NSI provides an estimation of the consistency of the local average values, high NSI indicating more inconsistent local averages [13]. In order to characterize the potential persistent/anti-persistent pattern (auto-correlation) present in the series, we used scaling exponents (α) computed by Detrended Fluctuation Analysis [1], [2], [21]. Alpha-values obtained in our previous study [21] were used. An overall view of the variables included in the statistical analyses is shown in table 1.

**Table 1:** Summary of the gait parameters.

Abbreviation	Definition	Formulae	Comments
M-SpT	Average values of the times series of the gait parameters, i.e. Step Time (SpT), Step Length (SpL), Step Speed (SpS = SpL/SpT) and Step Width (SpW)	<SpT>, N = 200	See fig. 1 and 2.
M-SpL		<SpL>, N = 200	
M-SpS		<SpS>, N = 200	
M-SpW		<SpW>, N = 200	
SD-SpT	Fluctuation magnitude (SD) of the times series of the gait parameters	SD(SpT), N = 200	See fig. 3
SD-SpL		SD(SpL), N = 200	
SD-SpS		SD(SpS), N = 200	
SD-SpW		SD(SpW), N = 200	
NSI-SpT	NonStationarity Index (NSI). Fluctuation of local standardized averages	SD(<SpT>loc), N = 20	See fig. 4
NSI-SpL		SD(<SpL>loc), N = 20	
NSI-SpS		SD(<SpS>loc), N = 20	
NSI-SpW		SD(<SpW>loc), N = 20	
α-ST	DFA results: Scaling exponent in times series of Stride Time (ST), Stride Length (SL) and Stride Speed (SS = SL/ST)	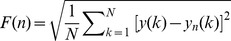 F(*n*) ∼ n^α^	Results from [21] Used in correlation analyses (fig. 5)
α-SL			
α-SS			

The table summarizes the gait parameters analyzed in the study.

### Statistical analysis

**Figure 1:**
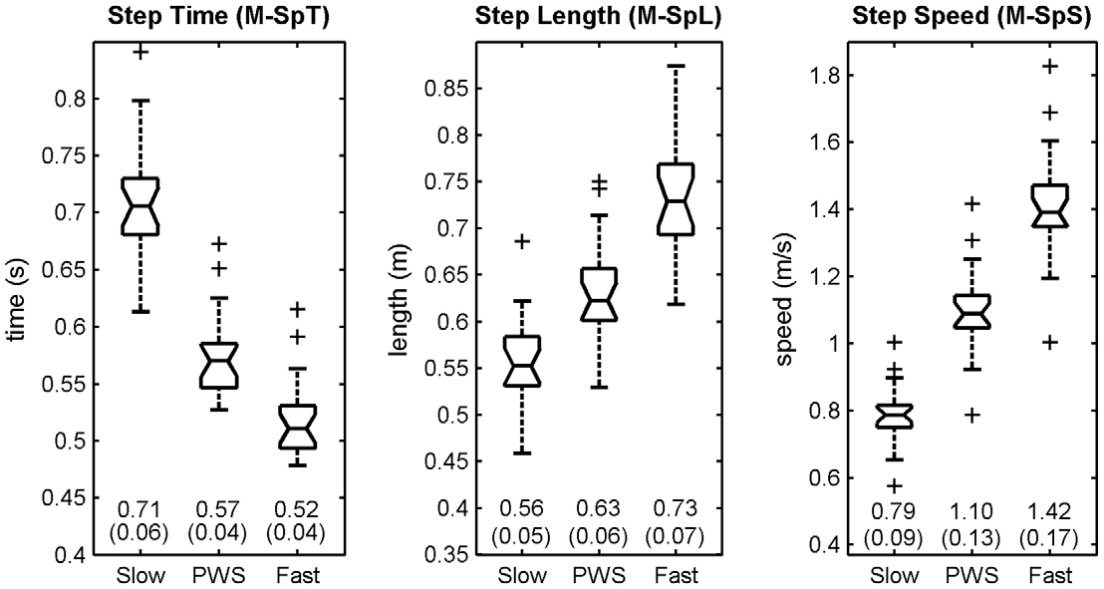
Basic gait parameters. Twenty healthy subjects walked 3×5 min. on a motorized treadmill. 100 strides (200 steps) were analyzed. Step Ttime (M-SpT i.e mean duration of the 200 steps), Step Length (M-SpL, i.e. mean length of the 200 steps) and Step Speed (M-SpS, i.e. mean speed of the 200 steps) are shown. The selected speeds were Preferred Walking Speed (PWS), 0.7x PWS (Slow) and 1.3x PWS (Fast). The range of individual results (N = 20) is presented with notched boxplots. Printed values are mean(SD).

**Figure 2:**
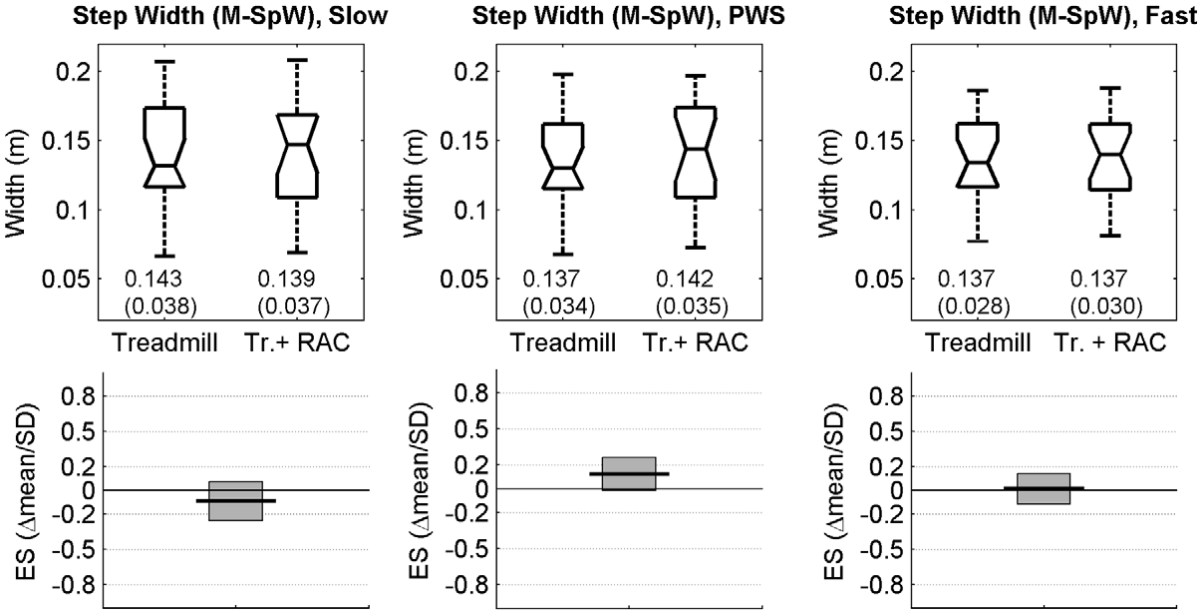
Step Width. Twenty healthy subjects walked 6×5 min. on a motorized treadmill without (Treadmill) and with Rhythmic Auditory Cueing (Tr. + RAC) at their preferred cadence for the given speed. 100 strides (200 steps) were analyzed. Step Width (M-SpW) is the mean of widths between consecutive steps (N = 200). Selected speeds were Preferred Walking Speed (middle, PWS), 0.7x PWS (left, Slow) and 1.3x PWS (right, Fast). The range of individual results (N = 20) is presented with notched boxplots. Printed values are mean(SD). Bottom panels show the Effect Size (ES, Hedge's g, variant of Cohen's d) of the auditory cueing, i.e. the mean difference normalized by SD. Boxes are the 95% confidence intervals for the effect size estimations.

**Figure 3:**
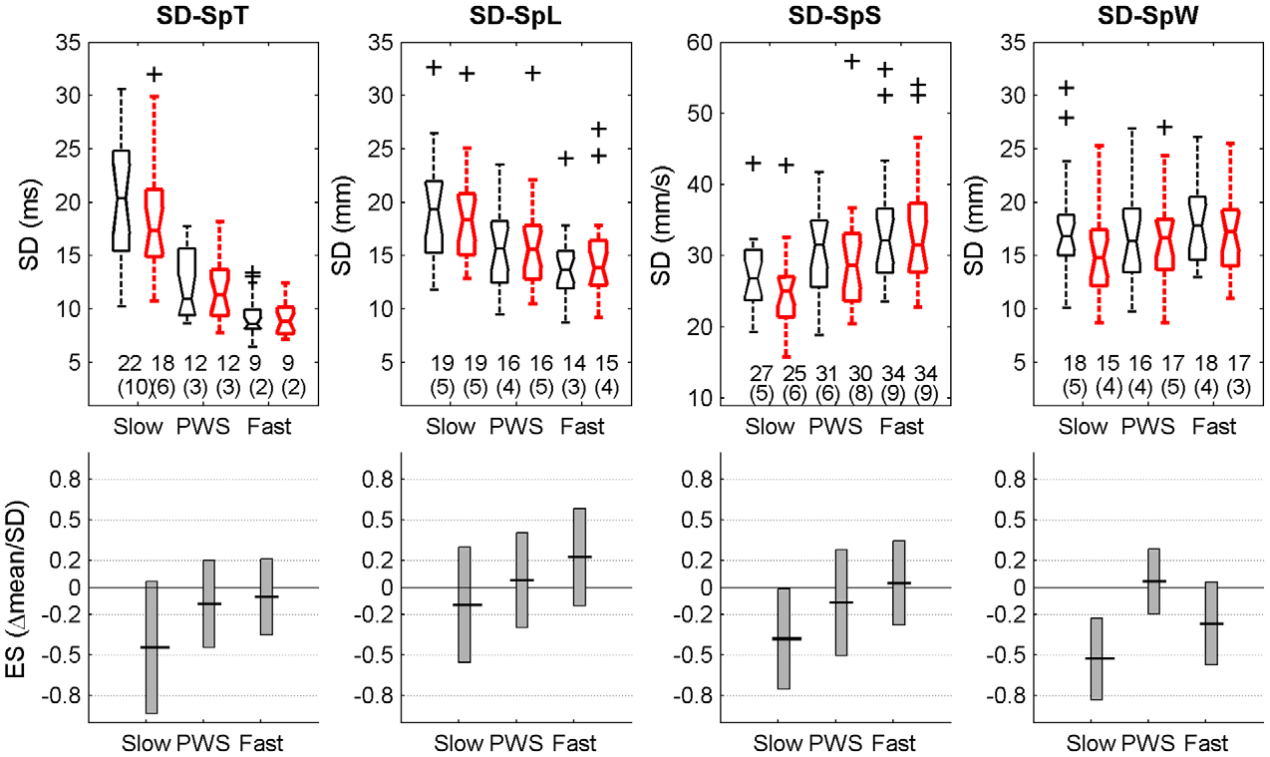
Step-to-step variability. Twenty healthy subjects walked 6×5 min. on a motorized treadmill without (left, black) and with Rhythmic Auditory Cueing (RAC, (metronome), right, red) at their preferred cadence for the given speed. 100 strides (200 steps) were analyzed. Variability indexes are Standard Deviations (SD, N = 200) of step time (SD-SpT), step length (SD-SpL), step speed (SD-SpS), and step width (SD-SpW). Selected speeds were Preferred Walking Speed (middle, PWS), 0.7x PWS (left, Slow) and 1.3x PWS (right, Fast). The range of individual results (N = 20) is presented with notched boxplots. Printed values are means (SD). Bottom panels show the effect size (Hedge's g, variant of Cohen's d) of the auditory cueing, i.e. the mean difference normalized by SD. Vertical boxes are the 95% confidence intervals for the effect size estimations.

**Figure 4:**
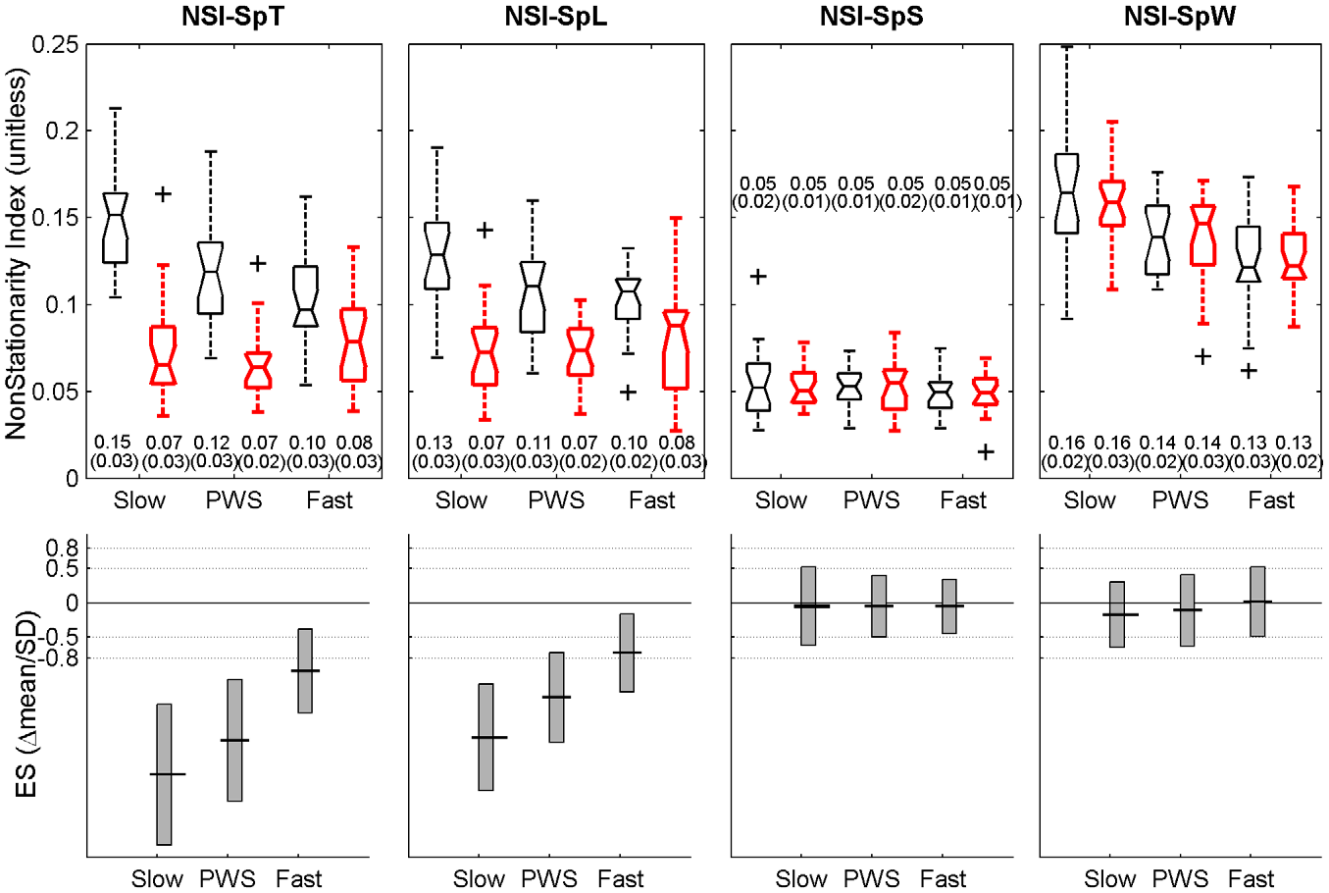
NonStationarity Index. Twenty healthy subjects walked 6×5 min. on a motorized treadmill without (left, blackl) and with Rhythmic Auditory Cueing (RAC, (metronome), right, red) at their preferred cadence for the given speed. 100 strides (200 steps) were analyzed. NonStationarity Index (NSI) is the variability (SD, N = 20) of the local means computed over 10 consecutive steps. Variables are step time (NSI-SpT), step length (NSI-SpL), step speed (NSI-SpS), and step width (NSI-SpW). Selected speeds were Preferred Walking Speed (middle, PWS), 0.7x PWS (left, Slow) and 1.3x PWS (right, Fast). The range of individual results (N = 20) is presented with notched boxplots. Printed values are mean(SD). Bottom panels show the effect size (Hedge's g, variant of Cohen's d) of the auditory cueing, i.e. the mean difference normalized by SD. Vertical boxes are the 95% confidence intervals for the effect size estimations.

 For each speed (low, PWS, high) and condition (without and with RAC), descriptive statistics consisted in notched boxplots (median and quartiles of the sample, fig. 1, 2, 3, 4). In addition, means and SD (N = 20 participants) are presented. The effect of RAC was assessed by using standardized Hedge's g: this standardized Effect Size (ES = delta(mean)/SD) is a modified version of the Cohen's d for inferential measure [24], [25]. The precision of the effect sizes was estimated with 95% Confidence Intervals (CI). CI were ±1.96 times the asymptotic estimates of the standard error of g. Graphical representation of ES and corresponding CI are given for each variable (see table 1) and speed condition, except for scaling exponents, which were already shown in our previous study [21]. Arbitrary thresholds for small (0.2), medium (0.5) and large (0.8) effects are also shown to ease the interpretation. It is worth noting that no effects of RAC were expected for average SpT, SpL and SpS, because, by design, the participants performed the two conditions at identical speed (imposed by the treadmill) and cadence (imposed by the metronome), what induced an identical SpL. As a result, descriptive statistics are given only for the “treadmill only” condition (fig. 1).

**Figure 5:**
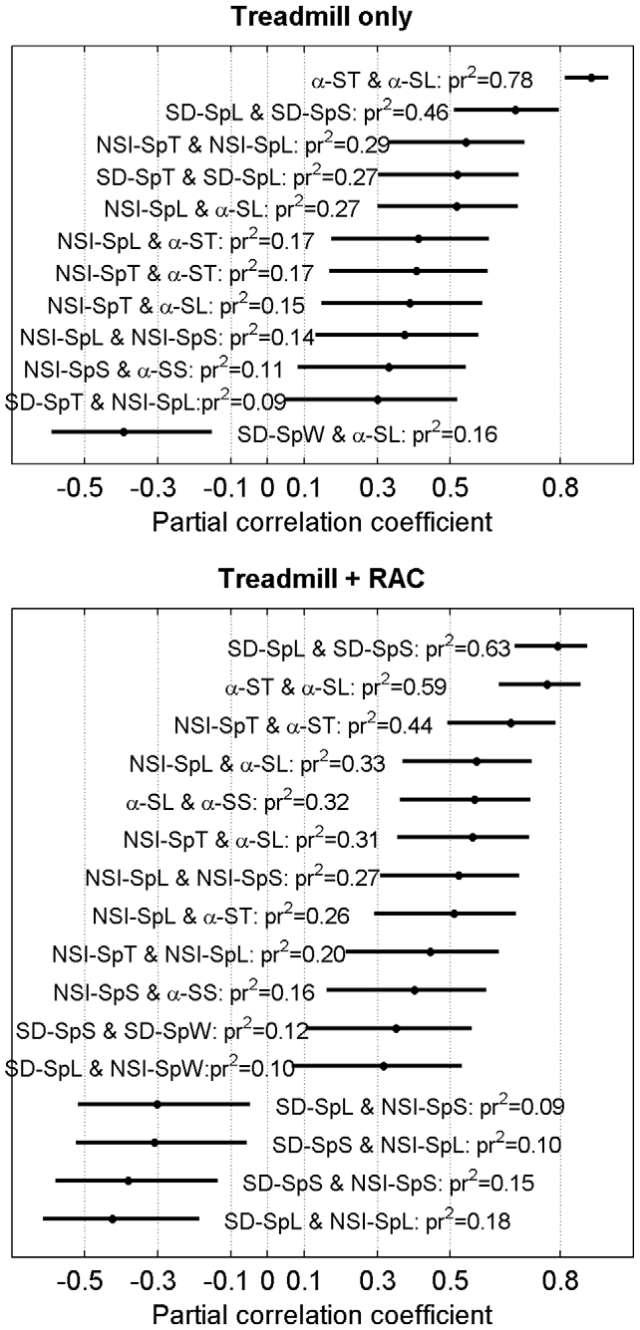
Partial correlations among gait variability indexes, controlled for walking speed. Partial correlation coefficients (pr) were computed among the 55 pairwise combinations of variability indexes (SD, NSI, α, see table 1). Pr's were assessed separately for treadmill only (top, N = 60), and treadmill + Rythmic Auditory Cueing (RAC, bottom, N = 60) conditions. Only pr absolute values greater than 0.3 are shown. Results are classified in decreasing order from top to bottom. Pr's were calculated by systematically taking speed (i.e. M-SpS) as the controlled variable. Horizontal continuous lines indicate 95% Confidence Intervals (CI) of the pr estimations. The definition of the abbreviations is presented in table 1. Pr^2^ are numeric values of the Coefficient of Determination, i.e. the percentage of variance explained by the linear regression model.

The strength of the association among variability indexes (SD, NSI, α), taking into account a potential speed effect, was estimated by computing partial Pearson's correlation coefficients (pr). The partial correlation between two variables x and y, controlling for a third one (z), is numerically equivalent to the correlation between the residuals from the regression of x and z and the residuals from the regression of y and z [24]. Thus, pr removes the variance explained by the third variables (z) from the variables of interest (x and y) and then quantifies the remaining correlation [24]. Practically, M-SpS was systematically controlled as the third variable (z) in pr computation: the result can be interpreted as correlation between variables holding speed constant. We also assessed the 95% CI based on an asymptotic normal distribution of 0.5*log((1+pr)/(1−pr)), with an approximate variance equal to 1/(N-4), N = 60. Given the eleven variability variables (table 1), 55 pairwise combinations were possible (11!/(2! × (11–2)!)  = 55). We computed pr separately for both conditions (treadmill only and RAC). We arbitrarily chose to present only correlation with |pr|>0.3, a threshold which corresponds to an association of moderate strength, according to Cohen [25] (fig. 5).

## Results

In order to ease the understanding of figures, the reader will find in table 1 a summary of the measured variables and their abbreviations.

Figure 1 presents the basic gait parameters measured in the 20 participants. By experimental design, the values are the same for both conditions (treadmill only, and treadmill + RAC). As expected, the increase in SpS induced a concomitant increase in SpL and decrease in SpT, because SpS = SpL/SpT. The inter-individual variability, expressed as CV (N = 20), was M-SpT: slow 8%, PWS 7%, fast 8%; M-SpL: slow 9%, PWS 10%, fast 10%; M-SpS: slow 11%, PWS 12%, fast 12%.

Step width was not constrained between conditions by the experimental protocol, and hence could be influenced by RAC. Figure 2 shows the extent of the results among participants, as well as the Effect Size (ES) of RAC. A substantial inter-individual variability was observed, ranging from 21% (fast speed) to 27% (slow speed). No RAC effect was found, with small and not significant ES.

Figure 3 shows the results for variability among gait parameters. Comparing the notched box, speed seemed to have a high impact on SD-SpT, but a moderate (SD-SpL, SD-SpS) or low (SD-SpW) effect on the other parameters. A high inter-individual variability is observed, which ranged from 19% (normal, slow, SD-SpS) to 45% (normal, Slow, SD-SpT). With one exception (SD-SpW, slow ES = −0.53), RAC did not produce a significant effect on gait variability. However, medium effect size are observed at slow speed for SD-SpT (ES = −0.45) and SD-SpS (ES = −0.38).

Results for NonStationarity Index (NSI) are presented in figure 4. Local fluctuations of speed (NSI-SpS) seemed not influenced by RAC, with low NSI (0.05) at every imposed speed and ES close to zero. In the same manner, no RAC effect was observed for step width (NSI-SpW), but higher values (0.13–0.16) and speed dependence are noted. Conversely, a very strong effect of RAC on step length (NSI–SpL) and step time (NSI–SpT) is evident (ES >>0.8). Under dual constraints conditions (treadmill + RAC), NSI remained rather low (0.07–0.08) for both SpL and SpT under every speed conditions. In contrast, higher NSI values (0.10–0.15) and speed dependence are evident in the treadmill only condition.

The figure 5 synthesizes the results of partial correlations (normalized for speed) among analyzed variables. Among 55 possible combinations, 11 and 16 partial correlation coefficients (pr) were higher than 0.3 in respectively treadmill only and treadmill + RAC conditions. A strong association was observed between scaling exponents of stride length and stride time under both conditions (α-ST vs. α-SL: pr = 0.88 and pr = 0.77). Other outstanding relationship concerns step length and speed variability (SD-SpL vs. SD-SpS: pr = 0.68 and pr = 0.79). It is also worth noting that local stationarity (NSI) seems moderately correlated with scaling exponents (α), with pr ranging from 0.33 to 0.66 among the different comparisons. Conversely, step width variability exhibited only few relevant (moderate) associations with other variables: treadmill only: SD-SpW vs. α-SL, pr = −0.4; treadmill+RAC: SD-SpS vs. SD-SpW, pr = 0.35; SD-SpL vs. NSI-SpW, pr = 0.32.

## Discussion

The present study is the follow-up of a previous study concerning persistent and anti-persistent pattern in gait fluctuations [21]. In this previous study, we observed that treadmill induced anti-persistent dynamics (α<0.5) in the time series of stride speed, but preserved the persistence (α>0.5) of stride time and length. On the contrary, all the three parameters were anti-persistent under dual-constraints (treadmill+RAC) condition. We suggested, according to the results and the previous literature, [1], [23], [26], [27], that two modalities of gait control co-exist: 1) a more automated/unconscious mode, which produces persistent, fractal-like pattern across numerous successive strides, what is likely related to the redundancy among the gait parameters to achieve steady gate; 2) a more voluntary/conscious mode relying upon fast over-correction of deviations in the controlled variable, which produces anti-persistent pattern among successive strides. In addition, the combination of two constraints (time and speed) induces anti-persistence in the three gait variables, what is likely the result of the cross-regulation of stride length and stride time and the absence of redundancy among the gait parameters to achieve the dual goal function.

Based on the same raw data, the present study explore in more details different variability indexes and their inter-relationships. To summarize the results, we observed that variability of the local means of gait parameters, expressed as Non-Stationarity index (NSI), was responsive to RAC in a similar was as scaling exponent described in our previous study [21]. Furthermore, we found that step width was not modified by RAC. In addition, RAC did not significantly change the variability (expressed as SD) of gait parameter, except for Step width variability at low speed. Concerning correlation results, we found a strong association between scaling exponents of stride length and stride width, as well moderate correlations between NSI and scaling exponents.

### Methodological considerations

The main methodological difference between this study and our previous study was that, here, we analyzed step-to-step variability, as compared to stride-to-stride fluctuations in the previous study. Furthermore, we used a constant number of 200 steps for the computation of variability index, as compared to the use of the full 5 min. time series in the previous study (360 to 626 steps). Auto correlation properties of time series, such as statistical persistence or long-range correlations as evidenced by DFA, are typically analyzed over several hundred consecutive points. Some studies used up to 1000 consecutive strides [1], [16]. Five minutes seems already a very short duration to capture reliably the scaling properties of the signal. On the other hand, research in the potential use of RAC in the rehabilitation of neurological disorders [19] requires experimental procedures that are adapted for patients with poor gait capacities and reduced gait perimeter. Therefore, the choice of smaller samples to analyze variability properties of the gait was made in the perspective to design future experiments with short walking tests (1–2 min) in gait disabled patients: because we observed that both NSI and scaling exponent respond similarly to RAC (see below for further explanation), it is likely that NSI would be useful in this perspective.

As we already mentioned in our previous study [21], the experimental design was not aimed at thorough analysis of speed effects. The design was build in order to ensure that the potential auditory cueing effect was not limited to PWS, but was also acting at slow and high speed. It has been observed that scaling exponents exhibited quadratic dependence (U-shaped form) as a function of speed [28]. Step time variability decrease exponentially or quadratically with speed [29], [30], [31]. It has also been suggested that slow speed alters gait pattern as compared to PWS (higher walk ratio [3]). Such non linear dependences and threshold effects cannot be satisfactorily analyzed with only 3 different speeds. As a result, we did not perform specific analysis of speed effects. In short, our results (fig. 3) confirm that low speed induces higher step time variability [3], [28], [29], [30], [32]. Moreover, speed seemed to have no significant effect on step width (fig. 2) and step width variability (fig. 3), what is in line with previous studies [30].

### Non Stationarity Index

A process is stationary when its statistical properties are time invariant. Stationarity of gait parameters, and especially stride time, has been extensively studied with various methodologies [13], [33], [34], [35]. It has been reported that the major source of non-stationarities in time series of stride time could be attributed to a change in the mean over time [34]. Consequently, we focused on mean fluctuations by using the NonStationarity Index (NSI). NSI is a simple measure of the consistency of the local average values, independent of the fluctuation magnitude of the original time series. Higher NSI values indicate more inconsistent local averages [13]. We observed (fig. 4) that NSI was highly responsive to RAC, in a similar way as scaling exponents [21]. It is very likely that the switch to a more conscious/voluntary mode of gait control not only produced anti-persistent dynamics [21], [23] but also less variable local means. This result is in accordance with the GEM (Goal Equivalent Manifold) model developed by Dingwell and Cusumano [23] based on redundancy theory. Motor redundancy refers to the fact that the motor control has numerous alternatives to perform a given task. It is thought that motor control allows high variability (more freedom) to parameters, which do no affect the desired value of the variable. On the contrary, it restricts the variability of parameters that are essentials for achieving the task [36], [37]. Accordingly, in order to maintain the constant speed imposed by the treadmill, a walking individual could choose an infinite combination of stride time and stride length. As a result, statistical persistence and less locally stable means (high NSI) are observed in step time and length fluctuations, because there is a redundancy among these parameters in achieving a given speed. Conversely, statistical anti-persistence and more locally stable means (low NSI) are observe in speed fluctuations. When treadmill and auditory cueing are combined, a degree of freedom is lost: the redundancy between length and time regulation disappears and the three parameters (time, length, speed) exhibit statistical anti-persistence [21] and more locally consistent means (low NSI).

### Step width

Regarding step width, modeling approach demonstrated that active lateral stabilization is needed to ensure stable walking, whereas gait is passively stable in the sagittal plane [8]. It has been suggested that lateral stabilization require high-level integration of sensory information, whereas passive stability in antero-posterior direction require far less sensing and control [8], [9]. Our results seem to indicate that RAC had no effect on average step width (M-SpW, fig. 2). While participants controlled their steps to be in time with the metronome, they did not significantly modify the lateral placement of the feet as compared to the “treadmill only” situation. Similarly, RAC did not change local mean variability of step width (identical NSI, fig. 4, right); however, higher values than for the other parameters (SpT, SpL, SpS) were observed. The only significant change (ES = −0.53) concerned step width variability under low speed condition (SD-SpW, fig. 3 right), that could be induced by the particularity of slow walking [3], [21]. It is likely that active balance control in the frontal plane –which results in optimized lateral foot placement– is independently regulated from the control in the sagittal plane (step length): in order to follow speed (treadmill) and time (metronome) constraints, individuals seem able to perform complex gait regulation in the antero-posterior direction, implying visual, auditory and proprioceptive integrative processes, with no concomitant change in lateral control.

### Step-to-step variability

It is very likely that motor control maintains a low magnitude of step-to-step variability in order to ensure minimal energy expenditure and optimal stability [6], [7], [8]. In overground, free walking, low variability (>3% CV) has been observed in SpT, SpL, SpS, even in long duration walking (30 min) [1]. In this previous study, no difference between free walking and metronome walking was observed regarding variability. Conversely, in our recent study [21], we showed that RAC markedly reduced stride to stride variability of stride time at low speed, with lower effect at higher speeds. We attributed this “low speed” effect to the high variability induced by slow walking [29]: it is likely that slow walking induces a specific spatial and temporal adaptation (higher walk ratio [3]) that was “un-natural” for many subjects, what induced a larger variability. The reference offered by RAC allowed individuals to reduce this variability. The present results did not confirm the RAC effect on gait variability. A general trend to lower variability with RAC was observed, but the large incertitude on ES values produced inconclusive results (fig. 3). The difference between the present analysis and the previous one is that step-to-step variability was analyzed rather stride-to-stride. Moreover, shorter samples (200 steps) were used. Although further studies are needed on this topic, it is evident that the fluctuation magnitude (CV or SD) are by far less responsive to RAC than scaling exponents or NSI. In response to the need of tighter gait control, it is likely that motor control seek to maintain low average fluctuation magnitude while step-to-step fluctuation dynamics is altered (as evidenced by scaling exponents or NSI).

### Correlations

In view of the numerous combinations (55) among the analyzed parameters (risks of type I statistical errors), and of the potential non-linear speed effects, caution is required in the interpretation of the partial correlations results (fig. 5). Therefore we limit the discussion to strong associations that seems physiologically grounded and that are coherent with our preliminary assumptions (GEM model).

In the frame of the GEM model [23], phase-randomized surrogate data analysis [21], [23] suggests that both stride length and stride time are simultaneously controlled (cross-regulated) stride after stride to ensure stride speed that match treadmill speed. Here, we showed a strong association between scaling exponents of stride time and stride length (α-ST vs. α-SL: pr = 0.88 and pr = 0.77): individuals exhibiting a high α-ST have a high probability to exhibit also a high α-SL value. In other words, deviations are allowed to persist (or to “anti-persist” under dual constraint conditions) in a similar way for both stride time and stride length. Therefore, this confirms that persistence characteristics are likely the result of the simultaneous, coordinated spatial and temporal regulation of the gait at each stride.

Corroborating the hypothesis that associates redundancy, statistical persistence and stationarity (see above), significant positive correlations between NSI and scaling exponents under both conditions were observed (NSI-SpT vs. α-ST: r^2^ = 0.17, r^2^ = 0.44. NSI-SpL vs. α-SL: r^2^ = 0.27, r^2^ = 0.33): when motor control allows deviations to persist (high α), local means exhibit higher variability (high NSI); accordingly, a more anti-persistent pattern (low α) is associated with more consistent local means (low NSI).

Step width variability (SD-SpW) and stationarity (NSI-SpW) exhibited only one moderate association under treadmill only condition (SD-SpW vs α-SL, pr = −0.40), and two moderate positive under treadmill + RAC conditions (SD-SpS vs. SD-SpW, pr = 0.35; SD-SpL vs. NSI-SpW, pr = 0.32). These inconsistent relationships tend to confirm that a lateral foot placement and antero-posterior gait modulations (i.e. SpL and SpT) are separately regulated.

### Conclusion

Externally cued walking is extensively studied because it exhibits positive effects on various gait characteristics of neurologically impaired patients, such as patients with Parkinson's disease [19], hemiparesis [38], or stroke [39]. RAC might be efficient because they would stimulate intact auditory-motor system, which would substitute to impaired gait control [40]. Furthermore, it has been shown that treadmill training also produced improvement in motor performance and ambulation in patients with Parkinson's disease [41]. It has been suggested that the treadmill (i.e. speed cueing) may be acting as an external cue to enhance gait rhythmicity and reduce gait variability [42]. As evidenced by our results, it is likely that the same type of mechanism is acting in healthy individuals: by activating alternative sensory-motor processes as compared to free (overground) walking, treadmill and RAC induced specific changes in step-to-step fluctuations. As far as we know, the simultaneous measurement of SpT, SpL and SpS and their fluctuation dynamics have never been performed in neurologically impaired patients. Future studies comparing the adaptation to external cues in patients with different pathologies would likely help to improve the use of cued walking in gait rehabilitation. The responsiveness of stationarity measure (NSI) to cued walking opens the perspective to perform shorter walking tests (1–2 min.) that would be adapted to patients with a reduced gait perimeter.
